# The Relationship Between Suboptimal Social Networks and Postoperative Delirium: The PNDABLE Study

**DOI:** 10.3389/fnagi.2022.851368

**Published:** 2022-06-13

**Authors:** Xinhui Tang, Hui Yv, Fei Wang, Jiahan Wang, Siyv Liu, Xiaoyue Wu, Rui Dong, Xu Lin, Bin Wang, Yanlin Bi

**Affiliations:** ^1^Department of Anesthesiology, Qingdao Municipal Hospital Affiliated to Qingdao University, Qingdao, China; ^2^Department of Anesthesiology, Qingdao Eye Hospital of Shandong First Medical University, Eye Institute of Shandong First Medical University, Qingdao, China; ^3^Department of Anesthesiology, Drum Tower Hospital Affiliated to Nanjing University Medical School, Nanjing, China

**Keywords:** social networks, lifestyle, cerebrospinal fluid, mediating effect, postoperative delirium

## Abstract

**Background:**

Although it has been proven that social networks are related to cognition, studies are conducted to characterize the correlation between social networks and postoperative delirium (POD).

**Objective:**

We investigated whether suboptimal social networks are a risk factor for POD, and to verify whether different levels of intimacy in the same social relationship can affect the concentration of cerebrospinal fluid (CSF) biomarkers, such as amyloid-β (Aβ42), total tau (T-tau), and phosphorylated tau (P-tau), and the mediating role of CSF biomarkers between social network and POD in middle-aged and elderly Han people.

**Methods:**

Our study recruited 743 participants from The Perioperative Neurocognitive Disorder and Biomarker Lifestyle (PNDABLE) study. Confusion Assessment Method (CAM) was used to evaluate the incidence of POD and its severity was measured using the Memorial Delirium Assessment Scale (MDAS). The social networks were measured using self-reported questionnaires about social ties. Mann–Whitney *U* test, Logistic Regression and Independent-samples test were used for Statistical Analysis.

**Results:**

The incidence of POD was 20.7%. Mann–Whitney *U* test showed that the total score of the social network was associated with POD (*P* < 0.001). Independent-samples test showed that different levels of intimacy in the same social relationship were significantly associated with CSF POD biomarkers, and mediation analyses revealed that the association between suboptimal social networks and POD was partially mediated by T-tau (proportion: 20%), P-tau (proportion: 33%), Aβ42/T-tau (proportion: 14%), and Aβ42/P-tau (proportion: 15%).

**Conclusion:**

Having suboptimal social networks is a risk factor for POD in middle-aged and elderly Han people. CSF POD biomarkers can mediate the correlation between suboptimal social networks and POD, which is mainly mediated by tau protein.

**Clinical Trial Registration:**

www.chictr.org.cn, identifier ChiCTR2000033439.

## Introduction

Postoperative delirium (POD) is a state of acute delirium caused by dysregulation of basic neuronal activity secondary to systemic disorders, and is a type of delirium with typical characteristics of consciousness fluctuations, inattention, confusion and changes in consciousness level. It is a common complication of the central nervous system after surgery, most commonly seen 1 ∼ 3 days after surgery. POD can lead to more postoperative complications, higher mortality, longer hospital stay and higher hospitalization costs. Therefore, to uncover the risk factors of POD and accurately identify the high-risk population of POD has always been one of the hot topics in perioperative research.

Cerebrospinal fluid (CSF) biomarkers include β-amyloid 42 (Aβ42), total Tau (T-tau) and phosphorylated Tau (P-tau), which are the core biomarkers of postoperative delirium (POD). Associated with the development and progression of POD, reduced CSF Aβ42 levels were shown to be an independent predictor (Strozyk et al., 2003; Lin et al., 2021), and the deposition of amyloid Aβ42 in amyloid plaques was reciprocally elevated (Blennow et al., 2015; Cunningham et al., 2019). Increased T-tau levels reflect the intensity of axonal degeneration (Hesse et al., 2001; Bitsch et al., 2002; Riemenschneider et al., 2003; Hampel et al., 2010), while increased P-tau levels are associated with the amount of neurofibrillary tangles in the brain of AD patients (Tapiola et al., 2009). Therefore, high levels of T-tau or P-tau suggest neurodegeneration (Idland et al., 2017), and their peak changes are related to the severity of POD (Parker et al., 2021). In addition, decreased Aβ/tau ratio is associated with POD (Xie et al., 2014).

Social networks refer to a system of relatively stable relationship formed by interaction between individual members of the society. They focus on the interaction and connection between people, and the consequent impact on people’s social behavior. A social network is essentially a social structure composed of many nodes, usually represented by individuals or organizations. Hence, social networks represent the social relationships ranging from casual acquaintances to tightly knit family among people and organizations.

Recently, the influence of social networks on cognitive status of middle-aged and elderly people has caught much attention. Maintaining an active social lifestyle in later life may enhance cognitive reserve and benefit cognitive function (Evans et al., 2018; Siette et al., 2020). Studies have proved that social relationship with friends or neighbors is associated with a lower risk of functional decline, and is not affected by family support (Murata et al., 2017). A rich social network was associated with a reduction in various adverse health outcomes and a lower risk of death in older adults (Oremus et al., 2019; Zahodne et al., 2021). In contrast, poor social networks may increase the risk of neurodegenerative diseases. For example, in a 2021 study, Akhter-Khan et al. (2021) reported that poor social networks (e.g., chronic loneliness) were independent predictors of AD. However, there are only a few studies on the correlation between social network and Postoperative cognitive dysfunction (POCD) and POD, and the pathological mechanism of neurodegenerative diseases caused by social network remains unclear.

Our study aims to explore whether suboptimal social networks are a risk factor for POD, and to characterize the mediating role of CSF core proteins between social networks and POD. It is of great clinical significance to explore the possibility of using social networks as an independent predictor to identify people at high risk of POD, and to validate the feasibility of using social network as an entry point to reduce the incidence of POD.

## Materials and Methods

### Perioperative Neurocognitive Disorder and Biomarker Lifestyle Study

The Perioperative Neurocognitive Disorder and Biomarker Lifestyle study (PNDABLE) investigates the pathogenesis, risk factors, and biomarkers of perioperative neurocognitive disorders among the northern Chinese Han population. PNDABLE aims to identify lifestyle factors that may increase the risk of PND in the non-demented northern Chinese Han population and provide evidence for disease prevention and early diagnosis. This study has been registered in the Chinese Clinical Trial Registry (clinical registration number ChiCTR2000033439) and approved by the Ethics Committee of Qingdao Municipal Hospital. CSF samples from all participants were collected upon obtaining written informed consent from the patient or the legal representative.

### Included Participants

The Han Chinese patients undergoing unilateral total knee arthroplasty [no gender limitations, aged 50 ∼ 90, American Society of Anesthesiologist (ASA) I ∼ II] combined with epidural anesthesia were enrolled in the PNDABLE study at Qingdao Municipal Hospital. The exclusion criteria include: (1) preoperative MMSE score ≤ 23 points; (2) drug or psychotropic substance abuse, as well as long-term use of steroid drugs and hormone drugs; (3) pre-operative III–IV hepatic encephalopathy; (4) recent major surgery; (5) severe visual and hearing impairments; (6) abnormal coagulation function before surgery; (7) central nervous system infection, head trauma, multiple sclerosis, neurodegenerative diseases including AD (e.g., epilepsy, Parkinson’s Disease), or other major neurological disorders; (8) major psychological disorders; (9) severe systemic diseases (e.g., malignant tumors) that may affect CSF or blood levels of AD biomarkers including Aβ and tau protein; (10) family history of genetic diseases.

**FIGURE 1:**
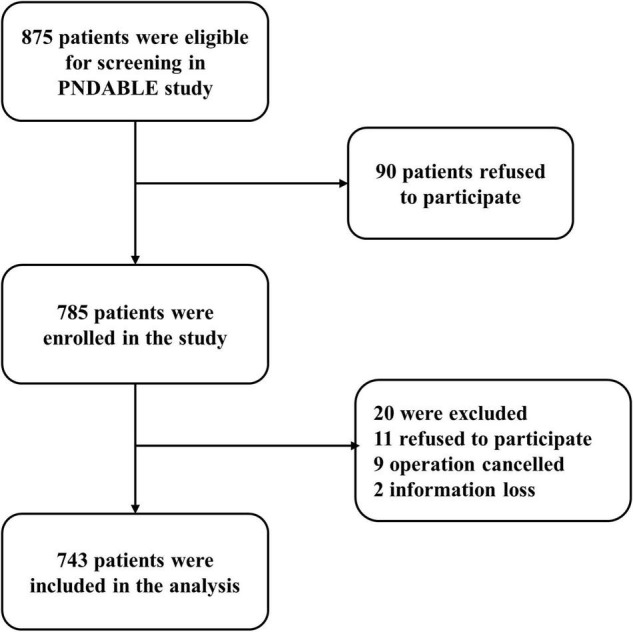
Flow diagram of PNDABLE study.

A total of 743 cognitively normal participants from PNDABLE had available information on covariates. According to whether POD occurred or not, the participants were divided into POD group and no postoperative delirium (NPOD) group. The patient recruitment flowchart is shown in Figure 1.

### Preoperative Assessment

We interviewed all the patients the day before surgery and collected their baseline data, including age, gender, body mass index (BMI), ASA physical status, years of education, smoking and drinking history and preoperative cognitive status. Other information including comorbidities and medical history was also collected according to the patients’ medical records. All the history collection, physical examination and dementia-related cognitive assessment were performed by an anesthesiologist.

### Anesthesia and Surgery

The participants did not receive preoperative medications, and they were instructed not to drink for 6 h and not to eat for 8 h before surgery. After entering the operating room, ECG, SpO2, and NBP were routinely monitored, venous access was opened, and 3 ml of whole venous blood was extracted. All patients underwent combined spinal-epidural block, with the lumbar spinous process space 3–4 (L3-4) being used as the puncture point. After successful puncture, 2 ml of CSF was extracted from the subarachnoid space, followed by injection of 2–2.5 ml Levobupivacaine (0.66%) for about 30 s. After anesthesia, the sensory level was controlled below the T8 level. During the surgery, oxygen was inhaled via mask at 5 L/min to maintain blood pressure within ±20% of the baseline value. If intraoperative NBP fell below 90 mmHg (1 mmHg = 0.133 kPa) or decreased by more than 20% of the baseline value, 5 mg ephedrine was injected intravenously. After the operation, the patient was sent to the post-anesthesia care unit (PACU).

### Measurements of Cerebrospinal Fluid Sampling

Cerebrospinal fluid samples were processed immediately within 2 h after standard lumbar puncture. Each sample was centrifuged at 2,000 × *g* for 10 min, and CSF samples were separated and stored in an enzyme-free EP (Eppendorf) tube (AXYGEN; PCR-02-C) at −80°C under the international BIOMARKAPD project for further use in this study.

Cerebrospinal fluid biomarkers of POD were measured by enzyme linked immunosorbent assay (ELISA) using the microplate reader (Thermo Scientific Multiskan MK3). Aβ42 (BioVendor, Ghent, Belgium Lot: No. 296-64401), P-tau (BioVendor, Ghent, Belgium Lot: QY-PF9092) and T-tau (BioVendor, Ghent, Belgium Lot: No. EK-H12242). All ELISA measurements were performed in strict accordance with the manufacturer’s instructions by experienced technicians who were blinded to the clinical information. The samples and standards were measured in duplicate, and the means of duplicates were used for the statistical analyses. All the antibodies and plates were from a single lot to exclude variability between batches. Moreover, the within-batch CV was <5% and the inter-batch CV was <15%.

### The Assessment of Social Networks

A quantitative assessment of the social network was conducted through a self-administered questionnaire and confirmed by people close to the patient (e.g., family members) (Ma et al., 2021). Patients were asked about how they got along with neighbors, relatives and friends to assess the degree of intimacy of different social relationships. The subjective options of intimacy included estrangement, moderate and intimacy, which were scored 0, 1, and 2, respectively. The evaluation was conducted using blind method, regardless of the evaluation and measurement results of other items from the same subject. The total score of the final social network is the sum of the intimacy score of the three social relationships, ranging from 0 to 6 points. The higher the score is, the closer the social relationship of the subject is.

### Neuropsychological Tests

The preoperative cognitive status was assessed by neurologists using the Mini-Mental State Examination (MMSE). Patients whose MMSE scores <23 points were excluded.

The delirium assessment was performed twice a day at 9:00–10:00 am and 2:00-3:00 pm on day 1–day 7 (or before discharge) post-operatively by an anesthesiologist. We used the visual analog scale (VAS) score of 0–10 (lower scores indicating lower levels of pain) to assess pain at the same time. POD was defined by the Confusion Assessment Method (CAM), and POD severity was measured using the Memorial Delirium Assessment Scale (MDAS). Thereafter, the post-operative CAM-positive and MDA-positive patients were recorded.

### Statistical Analysis

#### Sample Size Calculation

The preliminary test in this study found that 7 covariates were expected to enter the Logistic regression. The POD incidence was 10%. And the loss of follow-up rate was assumed to be 20%. Therefore, the required sample size was calculated to be 875.

#### Outcome Analysis

Demographic characteristics were summarized as median (p25 and p75) for continuous variables and number (column percentage) for categorical variables. The Kolmogorov–Smirnov test was used to determine whether the measurement data conformed to the normal distribution. The *t*-test (normally distributed data) or Mann–Whitney *U* test (skewed distribution) was performed for continuous variables, while chi-square tests for categorical variables. *P*-values were calculated for comparing the between-group difference.

Firstly, we took the total score of the social networks as a comprehensive factor to explore its relationship with POD. Univariate binary logistic regression analysis of POD, social network score, Aβ42, T-tau, P-tau, Aβ42/T-tau, and Aβ42/P-tau was performed. Age, gender, years of education, and MMSE were chosen as covariates in multivariate logistic regression analysis.

Secondly, we built two models to explore the robustness of the main results. The co-variables of the first model include age (continuous variable), sex (female = 0, male = 1), years of education (continuous variable), MMSE (continuous variable), history of alcohol consumption (no = 0, yes = 1), history of smoking (no = 0, yes = 1), history of diabetes (no = 0, yes = 1), history of hypertension (no = 0, yes = 1), history of coronary heart disease (no = 0, yes = 1). The co-variables of the second model include age (65–90 years), sex (female = 0, male = 1), years of education (continuous variable), MMSE (continuous variable), history of alcohol consumption (no = 0, yes = 1), history of smoking (no = 0, yes = 1), history of diabetes (no = 0, yes = 1), history of hypertension (no = 0, yes = 1), history of coronary heart disease (no = 0, yes = 1). The multivariable logistic regression analysis was performed.

Thirdly, we divided social networks into three subgroups. Independent-samples test was used to examine the relationship between scores of the three subcomponents and CSF biomarkers of POD.

Finally, to examine whether the association between social network and POD was mediated by CSF POD biomarkers, logistic regression models were fitted based on the methods. The first equation regressed the mediator (CSF POD biomarkers) on the independent variable (social network). The second equation regressed the dependent variable (POD) on the independent variable (social network). The third equation regressed the dependent variable on both the independent variable and the mediator variable. Mediation effects were established if the following criteria were simultaneously met: (1) social network were significantly related to CSF POD biomarkers; (2) social network was significantly or not significantly related to POD; (3) CSF POD biomarkers were significantly related to POD; and (4) the association between social network and POD was attenuated when CSF POD biomarkers (the mediator) were added in the regression model. Furthermore, the attenuation or indirect effect was estimated, with the significance determined using 10,000 bootstrapped iterations, where each path of the model was controlled for age, gender, years of education, and MMSE.

The data were analyzed using SPSS version 23.0 (SPSS Inc., Chicago, IL, United States), R software version 4.4.1(R Foundation for Statistical Computing, Vienna, Austria) and Stata MP16.0 (Solvusoft Corporation Inc, Chicago, IL, United States). *P*-value < 0.05 was considered significant except where specifically noted.

## Results

### Participant Characteristics

We recruited 875 participants, of which 743 met the requirements of our study and 132 were excluded. The reasons for dropping out were shown in Figure 1. Of the enrolled patients, 154 subjects experienced POD within 7 days after operation or before discharge, therefore, the incidence of POD was 20.7%. Participants were divided into two groups based on the presence or absence of POD (POD group and NPOD group). The demographic and clinical data of two groups were summarized in Table 1 (Pinho et al., 2016; Wang et al., 2018).

**TABLE 1:** Characteristics of included participants in PNDABLE database.

Characteristics	All participants(*n* = 743)		*P*-value*
	POD(*n* = 154)	NPOD(*n* = 589)	
Age, year	74(71–78)	59(51–65)	<0.001***
Female, yes (%)	60(39.0)	236(40.0)	0.835
Education, year	9(6–12)	9(9–12)	0.072
MMSE	28(27–29)	29(28–30)	<0.001***
MDAS	12(3–22.25)	1(1–7)	<0.001***
Hypertension, yes, no. (%)	72(46.8)	190(32.2)	0.001**
Diabetes mellitus, yes, no. (%)	41(26.6)	82(13.9)	<0.001***
CHD, yes, no. (%)	35(22.7)	61(10.4)	<0.001***
Smoking history, yes, no. (%)	38(32.7)	175(29.7)	0.231
Drinking history, yes, no. (%)	42(37.5)	211(35.8)	0.056
**CSF biomarkers, pg/ml**			
Aβ42	245.0(142.8–405.3)	350.1(231.7–492.9)	<0.001***
T-tau	277.9(199.1–609.6)	190.7(144.8–260.1)	<0.001***
P-tau	63.2(40.2–83.0)	36.5(28.1–46.3)	<0.001***
Aβ42/T-tau	0.8(0.4–1.7)	1.8(1.1–2.7)	<0.001***
Aβ42/P-tau	4.4(2.0–8.2)	9.2(6.1–13.8)	<0.001***
Social network score	4(3–6)	6(4–6)	<0.001***

*P-value < 0.05, **P-value < 0.01, ***P-value < 0.001. CSF, cerebrospinal fluid; Aβ, amyloid-β; T-tau, total tau; P-tau, phosphorylated tau; CHD, Coronary Heart Disease; MMSE, Mini-Mental State Examination (Score range: 24–30); MDAS, Memorial Delirium Assessment Scale; Social network score (Score range: 0–6). Data are presented as median (IQR) unless otherwise indicated.

### Correlation Analysis of Social Network Score and Cerebrospinal Fluid Biomarkers With Postoperative Delirium

**FIGURE 2:**
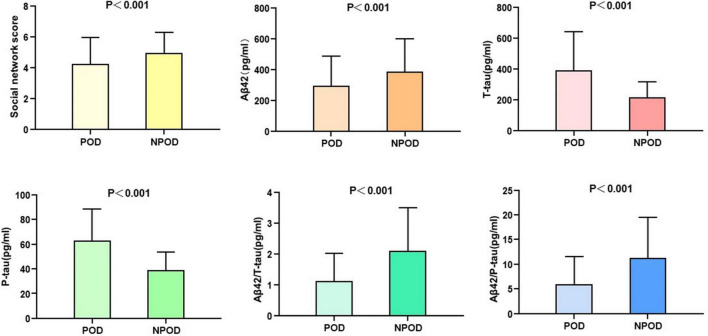
Social network score and expression of biomarkers in CSF of POD patients and NPOD controls.

Mann–Whitney *U* test showed that social network score and CSF biomarkers (Aβ42, T-tau, P-tau, Aβ42/T-tau, and Aβ42/P-tau) were associated with POD (*P* < 0.001) (Table 1). The social network score and the CSF levels of Aβ42, Aβ42/T-tau, and Aβ42/P-tau in patients with delirium were significantly lower than in those without delirium. However, the CSF levels of T-tau and P-tau in POD patients were significantly lower than those in NPOD patients (Figure 2).

Univariate binary logistic regression analysis showed that increased CSF levels of T-tau (crude OR = 1.006, 95% CI 1.005–1.007, *P* < 0.001) and P-tau (crude OR = 1.062, 95% CI 1.051–1.074, *P* < 0.001) were risk factors for POD. However, increased levels of social network (crude OR = 0.723, 95% CI 0.642–0.814, *P* < 0.001), Aβ42 (OR = 0.997, 95% CI 0.996–0.998, *P* < 0.001), Aβ42/T-tau (crude OR = 0.407, 95% CI 0.325–0.510, *P* < 0.001), and Aβ42/P-tau (crude OR = 0.833, 95% CI 0.795–0.874, *P* < 0.001) were protective factors for POD (Table 2).

**TABLE 2:** Logistic regression analysis and sensitivity analysis in PNDABLE study.

	Model 1		Model 2		Model 3		Model 4	
	OR (95% CI)	*P*-value	OR (95% CI)	*P*-value	OR (95% CI)	*P*-value	OR (95% CI)	*P*-value
Social network score	0.723(0.642–0.814)	<0.001***	0.831(0.700–0.986)	0.034*	0.821(0.689–0.979)	0.028*	0.830(0.695–0.992)	0.041*
Aβ42, pg/ml	0.997(0.996–0.998)	<0.001***	0.998(0.997–1.000)	0.017*	0.998(0.997–1.000)	0.025*	0.998(0.997–1.000)	0.028*
T-tau, pg/ml	1.006(1.005–1.007)	<0.001***	1.005(1.00–1.007)	<0.001***	1.005(1.004–1.007)	<0.001***	1.005(1.003–1.007)	<0.001***
P-tau, pg/ml	1.062(1.051–1.074)	<0.001***	1.063(1.045–1.081)	<0.001***	1.064(1.046–1.082)	<0.001***	1.067(1.048–1.087)	<0.001***
Aβ42/T-tau, pg/ml	0.407(0.325–0.510)	<0.001***	0.544(0.408–0.724)	<0.001***	0.548(0.409–0.733)	<0.001***	0.552(0.412–0.739)	<0.001***
Aβ42/P-tau, pg/ml	0.833(0.795–0.874)	<0.001***	0.866(0.825–0.910)	<0.001***	0.867(0.825–0.910)	<0.001***	0.870(0.828–0.915)	<0.001***

*P-value < 0.05, **P-value < 0.01, ***P-value < 0.001. Aβ, amyloid-β; T-tau, total tau; P-tau, phosphorylated tau. Model 1: The unadjusted logistic regression. Model 2: Adjusted logistic regression, the adjustment factors include age, gender, years of education, and MMSE. Model 3: First sensitivity analysis was based on more covariates including age, gender, years of education, MMSE, history of alcohol consumption and smoking, diabetes, hypertension, coronary heart disease. Model 4: Second sensitivity analysis was based on selecting only individuals older than 65 years.

After adjustment for the age, gender, years of education and MMSE, similar results were: T-tau (crude OR = 1.005, 95% CI 1.00–1.007, *P* < 0.001) and P-tau (crude OR = 1.063, 95% CI 1.045–1.081, *P* < 0.001) remained as risk factors for POD. Social network score (crude OR = 0.831, 95% CI 0.700–0.986, *P* = 0.034), Aβ42 (OR = 0.998, 95% CI 0.997–1.000, *P* = 0.017), Aβ42/T-tau (crude OR = 0.544, 95% CI 0.408–0.724, *P* < 0.001), and Aβ42/P-tau (crude OR = 0.866, 95% CI 0.825–0.9110, *P* < 0.001) were still protective factors for POD (Table 2).

Furthermore, sensitivity analysis was performed to verify the stability of the results. We first added more covariates including age, gender, years of education, MMSE, history of alcohol consumption, history of smoking, diabetes, hypertension, and coronary heart disease into the multivariate logistic regression analysis. The results showed that the social network score (crude OR = 0.812, 95% CI 0.689–0.979, *P* = 0.028) was still correlated with POD. Then we analyzed whether the association would change if only individuals aged over 65 at the baseline were selected, and found that the social network score (crude OR = 0.830, 95% CI 0.695–0.992, *P* = 0.041) and CSF biomarkers remained stable across two sensitivity analyses in our study (Table 2).

### The Relationship Between Intimacy Degrees of Three Subcomponents and Cerebrospinal Fluid Postoperative Delirium Biomarkers

In the total population, social network subcomponents were significantly associated with CSF POD biomarkers. In the subcomponent of social relationships with friends, when intimacy degree changed from distant to close, the CSF levels of P-tau (*t* = 2.678, *P* = 0.011), Aβ42/T-tau (*t* = −2.163, *P* = 0.031), and Aβ42/P-tau (*t* = −2.291, *P* = 0.028) were also changed. In particular, when the intimacy degree changed from moderate to close, the CSF levels of P-tau (*t* = 2.376, *P* = 0.018) and Aβ42/P-tau (*t* = −2.013, *P* = 0.044) were significantly reduced.

In the subcomponent of social relationships with relatives, when the degree of intimacy changed from distant to moderate, the CSF level of Aβ42/T-tau was significantly increased (*t* = −3.226, *P* = 0.002). When the intimacy degree changed from distant to close, the CSF levels of T-tau (*t* = 2.078, *P* = 0.047), P-tau (*t* = 2.656, *P* = 0.008), Aβ42/T-tau (*t* = −4.386, *P* < 0.001), and Aβ42/P-tau (*t* = −2.253, *P* = 0.025) were also changed. When the intimacy degree changed from moderate to close, Aβ42 (*t* = −2.037, *P* = 0.042) and Aβ42/P-tau (*t* = −2.222, *P* = 0.027) were significantly increased.

**FIGURE 3:**
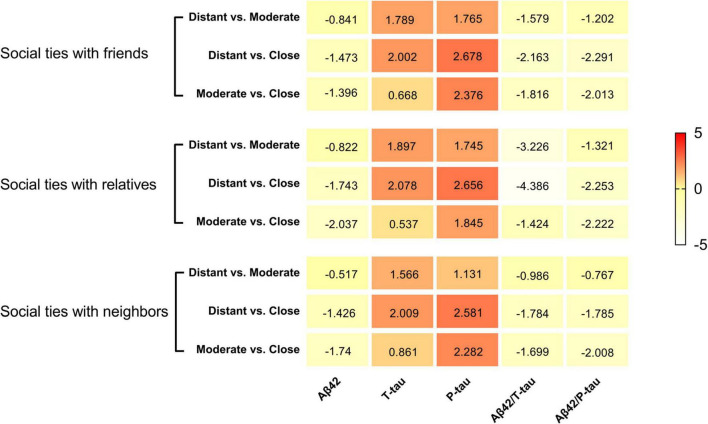
Heat maps for social networks and CSF biomarkers of POD. The social networks showed several suggestive or significant associations with CSF indicators of multiple pathologies of POD in the whole participants. The color-coding and the positive or negative number in each cell represent a comparison of mean values between the two groups inferred from the Independent-Samples *T*-Test.

In the subcomponent of social relationships with neighbors, the intimacy degree mainly affects the CSF levels of P-tau [distant versus close (*t* = 2.581, *P* = 0.010), moderate versus close (*t* = 2.282, *P* = 0.023)] and Aβ42/P-tau [moderate versus close (*t* = −2.008, *P* = 0.045)] (Table 3 and Figure 3).

**TABLE 3:** Three subgroups of social network and CSF biomarkers.

Subgroup	Aβ42 (63.5–998.0) pg/ml		T-tau (56.8–986.3) pg/ml		P-tau (10.1–130.4) pg/ml		Aβ42/T-tau (0.1–9.3)		Aβ42/P-tau (0.7–44.3)	
	*t*	*P*-value*	*t*	*P*-value*	*t*	*P*-value*	*t*	*P*-value*	*t*	*P*-value*
**Social ties with friends**										
Distant vs. Moderate	−0.841	0.401	1.789	0.082	1.765	0.085	−1.579	0.115	−1.202	0.230
Distant vs. Close	−1.473	0.142	2.002	0.053	2.678	0.011*	−2.163	0.031*	−2.291	0.028*
Moderate vs. Close	−1.396	0.163	0.668	0.504	2.376	0.018*	−1.816	0.070	−2.013	0.044*
**Social ties with relatives**										
Distant vs. Moderate	−0.822	0.412	1.897	0.067	1.745	0.082	−3.226	0.002**	−1.321	0.188
Distant vs. Close	−1.743	0.082	2.078	0.047*	2.656	0.008**	−4.386	<0.001***	−2.253	0.025*
Moderate vs. Close	−2.037	0.042*	0.537	0.591	1.845	0.066	−1.424	0.155	−2.222	0.027*
**Social ties with neighbors**										
Distant vs. Moderate	−0.517	0.606	1.566	0.119	1.131	0.259	−0.986	0.325	−0.767	0.444
Distant vs. Close	−1.426	0.155	2.009	0.051	2.581	0.010*	−1.784	0.075	−1.785	0.075
Moderate vs. Close	−1.740	0.082	0.861	0.390	2.282	0.023*	−1.699	0.090	−2.008	0.045*

*p-value < 0.05, **p-value < 0.01, ***p-value < 0.001. Aβ, amyloid-β; T-tau, total tau; P-tau, phosphorylated tau. Social ties between families, neighbors, and, relatives are not related.

### Causal Mediation Analyses

**FIGURE 4:**
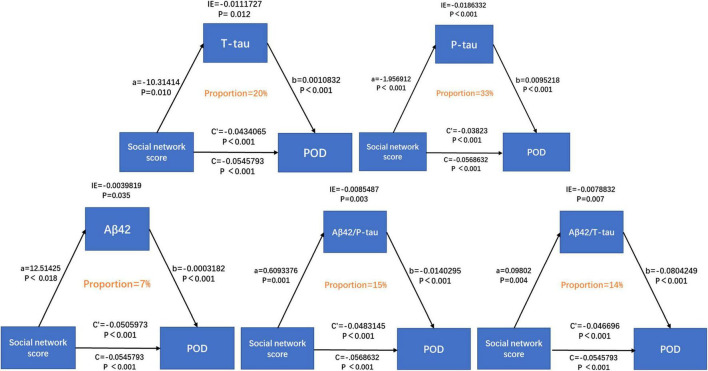
Mediation analyses.

We further explored the mediating effect of CSF biomarkers between suboptimal social networks and POD. The mediated effect analysis showed that CSF T-tau (proportion = 20%) and P-tau (proportion = 33%) contents as well as Aβ42/T-tau (proportion = 14%) and Aβ42/P-tau (proportion = 15%) had significant mediation effect on the correlation between suboptimal social networks and POD (Figure 4).

## Discussion

The results of this study support the association between suboptimal social networks and POD, with low social network score being an independent predictor. Social networks are founded upon interpersonal relationships, the establishment of which in turn may promote social participation and social support (Czaja et al., 2021). On the other hand, a low score for social networks may indicate relative alienation and even social isolation. In addition, animal models have proved that social isolation may cause or even aggravate memory loss (Leser and Wagner, 2015), while a rich social environment may reduce cognitive deficits in rats (Hsiao et al., 2012; Huang et al., 2015). A study conducted in 2020 demonstrated that social isolation is associated with cognitive decline in older adults (Yu et al., 2021), confirming the notion that high levels of social isolation may influence cognitive status in older adults by affecting episodic memory and mental states. Numerous studies have shown that a good social network can significantly improve the cognitive status of older adults (Fratiglioni et al., 2000, 2004; Balouch et al., 2019). People with rich social networks have more opportunities to communicate with the outside world, and are more likely to promote cognitive, physical and social participation, enhance physical and mental stimulation, and effectively reduce negative psychological states, thereby reducing the risk of cognitive dysfunction. Moreover, Preoperative cognitive status was related to POD (Choi et al., 2020), which is supported by our results. Besides, we could apply the results of this study to identify patients at a high risk for POD. Since POD, as a common perioperative complication in elderly patients, may affect the length of hospital stay and long-term prognosis, accurate preoperative identification of these patients and immediate intervention may reduce the incidence of POD, thereby shortening the length of hospital stay and improving long-term outcomes.

Based on the social network questionnaire, this study divided social networks into three subgroups. The scores of the three subcomponents reflected how close the patients were to different social relationships. Our study further examined the relationship between the scores of three subcomponents and CSF biomarkers of POD. The results confirmed that different social relationships are associated with different incidence of POD, and that the level of intimacy within the same social relationship affected CSF biomarkers.

Pathological changes of POD mainly include phosphorylation of tau protein and deposition of amyloid protein. In addition, it has been reported that the ratio between CSF biomarkers is more sensitive to the identification of amyloid deposition than individual biomarkers (e.g., Aβ42/Aβ40 vs. Aβ42) (Janelidze et al., 2016). Therefore, the ratio was also used in this study to explore the relationship between social network and POD, and it was concluded that the intimacy of the three social relationships mainly affected the incidence of POD by modulating the level of T-tau and P-tau proteins, Furthermore, the degree of intimacy with friends mainly affected the value of P-tau, Aβ42/T-tau, and Aβ42/P-tau, the degree of intimacy with neighbors mainly affected the value of P-tau, and, the degree of intimacy with relatives affected the value of Aβ42, T-tau, P-tau, Aβ42/T-tau, and Aβ42/P-tau.

In order to explore the mechanism of how social network relates to the incidence of POD, we explored the mediating effect of CSF biomarkers between social network and POD, and concluded that social network mainly affects T-tau protein, P-tau protein and the level of Aβ42/T-tau protein, Aβ42/P-tau affected the occurrence of POD. It has been found that rich social networks may reduce Aβ deposition and P-tau production (Huang et al., 2015; Donovan et al., 2016). In line with our study, previous research showed an elevated Aβ aggregation in isolated aged APP/PS1 mice, which was associated with increased secretase and decreased neprilysin expression (Hsiao et al., 2012; Huang et al., 2015). Besides, D’oleire Uquillas et al. (2018) found that after controlling for age, sex, and the Alzheimer’s disease genetic risk factor, apolipoprotein E (APOE), a higher tau pathology in the right entorhinal cortex was associated with greater loneliness. This is consistent with our research.

In this study, we established three correction models, four variables in Model 2 (age, sex, years of education, and MMSE) and nine variables in Model 3 (age, sex, MMSE, years of education, history of alcohol consumption, history of smoking, history of diabetes, history of hypertension, and history of coronary heart disease) were used as co-variables for regression analysis. Suboptimal social networks are still significantly correlated with the occurrence of POD. In addition, patients older than 65 years old were included in the regression analysis as co-variable of model 4, and the conclusion has not changed, indicating that our conclusion is robust— Suboptimal social networks is a risk factor for the occurrence of POD.

In addition to confirming the correlation between social network and the incidence of POD in the elderly population, this study is also consistent with the recent research hotspot of “age-friendly environment.” The concept of an age-friendly environment has attracted a lot of attention since its inception. How to integrate the advantages of medicine, environment, society and other disciplines and fully identify the environmental factors conducive to health and longevity is one of the cutting-edge research directions. Studies have proved that both natural and social environments have a significant impact on the physical and mental health and cognitive function of the elderly. For example, a study in 2021 confirmed that social isolation is harmful to the cognitive health of the elderly population (Li et al., 2021). Since the COVID-19 pandemic, home isolation has significantly increased the incidence of social isolation among the elderly, while unsurprisingly reducing their social support. Our result confirmed that the lack of social network is a risk factor for POD in aged population. Hence in future studies, through the preoperative score of social networks, we can predict or identify people at high risk of POD, make timely and necessary intervention, reduce the incidence of POD, so as to improve the long-term prognosis and promote the construction of healthy aging society.

There are some limitations to our study. Firstly, at the stage of data collection, we only counted three co-morbidities of patients, and the number of comorbid conditions was small. In subsequent studies, we will include as many comorbid conditions as possible to make the results more convincing. Secondly, this study is only a cross-sectional study which explores the relationship between preoperative total social network score and POD in middle-aged and elderly patients around the time of elective surgery, without following-up the incidence of delirium and cognitive function status of patients for a long time after surgery. Hence we will further explore in future studies the influence of suboptimal social networks and other social factors on longitudinal changes of POD neuropathology through multi-center CSF and neuroimaging tests with a large sample size. Finally, this study only focused on the effect of social network on POD among Chinese Han population. As different groups and regions have different cultural backgrounds, the identified effect of social network on the cognitive function and mental health status of the elderly may change. Therefore, it is necessary to sort out the dimensions of social networks, develop social network measurement tools suitable for specific cultural backgrounds, and explore the correlation between the different groups and regions of China with different cultural backgrounds.

## Conclusion

Our study confirms that suboptimal social networks are a risk factor for POD, and that different levels of intimacy in the same social relationship can affect the concentration of CSF POD biomarkers, mainly mediated by tau protein, hence influencing the incidence of POD. In other words, it can affect cognitive function by affecting the concentration of CSF biomarkers. This suggests that suboptimal social networks, as a risk factor for POD, can predict the population at high risk. This work will provide information for future social behavior research and age-friendly environment research, thus facilitating the development of intervention strategies for POD and promoting the construction of healthy aging environment.

## Data Availability Statement

The raw data supporting the conclusions of this article will be made available by the authors, without undue reservation, to any qualified researcher.

## Ethics Statement

The studies involving human participants were reviewed and approved by Chinese Clinical Trial Registry (clinical registration number ChiCTR2000033439) and approved by the Ethics Committee of Qingdao Municipal Hospital. The patients/participants provided their written informed consent to participate in this study.

## Author Contributions

YB conceived the current study. FW, JW, SL, and XW performed the experiments. RD, XL, HY, and BW analyzed the data. XT and BW performed the experiments and wrote and revised the manuscript. All authors have contributed to the manuscript revising and editing critically for important intellectual content and given final approval of the version and agreed to be accountable for all aspects of the work presented here and read and approved the final manuscript.

## Conflict of Interest

The authors declare that the research was conducted in the absence of any commercial or financial relationships that could be construed as a potential conflict of interest.

## Publisher’s Note

All claims expressed in this article are solely those of the authors and do not necessarily represent those of their affiliated organizations, or those of the publisher, the editors and the reviewers. Any product that may be evaluated in this article, or claim that may be made by its manufacturer, is not guaranteed or endorsed by the publisher.

## Abbreviations

AD: Alzheimer’s disease
POCD: Postoperative cognitive dysfunction
POD: postoperative delirium
NPOD: no postoperative delirium
CSF: cerebrospinal fluid
A β: β-amyloid
T-tau: total tau
P-tau: phosphorylated tau
CAM: Confusion Assessment Method
MDAS: Memorial Delirium Assessment Scale
ELISA: enzyme linked immunosorbent assay
MMSE: Mini-Mental State Examination
PACU: post-anesthesia care unit
ASA: American Society of Anesthesiologist.
